# Innovations in dementia screening: a systematic review and meta-analysis of virtual reality assessments

**DOI:** 10.3389/fpsyg.2025.1606562

**Published:** 2025-11-10

**Authors:** Åsmund Gausemel, Petra Filkuková

**Affiliations:** 1 Department of Psychology, University of Copenhagen, Copenhagen, Denmark; 2 Department of Psychology, University of Inland Norway, Lillehammer, Norway; 3 Simula Research Laboratory, Department of High Performance Computing, Oslo, Norway

**Keywords:** mild cognitive impairment, virtual reality, dementia, screening, artificial intelligence (AI), machine learning

## Abstract

**Background:**

The rising prevalence of dementia, driven by aging populations, presents a global public health challenge. Pharmacological treatments offer limited benefits unless initiated during pre-symptomatic stages, emphasizing the need for early, accurate, and cost-effective screening methods. This review investigates the diagnostic accuracy and feasibility of virtual reality-based assessments for identifying mild cognitive impairment, a prodromal stage of dementia.

**Methods:**

A systematic literature search of PubMed, PsycINFO, and IEEE Xplore was conducted to identify studies evaluating the diagnostic performance of virtual reality tools for mild cognitive impairment detection. Pooled sensitivity and specificity were calculated through meta-analysis, and methodological quality was assessed using the QUADAS-2 tool. The review adhered to PRISMA guidelines.

**Results:**

Twenty-nine studies met the inclusion criteria. Virtual reality-based assessments demonstrated pooled sensitivity and specificity of 0.883 and 0.887, respectively. Among 13 studies using machine learning, pooled sensitivity was 0.888 and specificity 0.885. Further sub-group analysis was done according to immersion degree, MCI-subtype and reference standard. Tools integrating machine learning with EEG or movement data showed particular promise.

**Conclusion:**

The findings of this meta-analysis indicate that virtual reality (VR) assessments have a promising level of accuracy for the detection of mild cognitive impairment (MCI). Nevertheless, the results are preliminary, and their interpretation warrants caution due to the substantial methodological heterogeneity observed among the included studies. Despite the potential of VR as a cost-effective solution for dementia screening, its implementation faces notable barriers, including the requirement for specialized personnel and the absence of clear data regarding software and support costs.

## 1 Introduction

The escalating global burden of dementia represents one of the most pressing health and societal challenges of the 21st century, with projections indicating a tripling of affected individuals by 2050 (Alzheimer Europe, 2019). Dementia is a general term for a decline in cognitive function severe enough to interfere with daily life and independence (Arvanitakis et al., 2019). It is not a specific disease but a syndrome caused by various underlying conditions that affect the brain (Arvanitakis et al., 2019). Aging is the primary risk factor for dementia, as its prevalence increases significantly with advancing age (Mecocci and Boccardi, 2021; Van Der Flier, 2005). While dementia is not a normal part of aging, the age-related changes that occur in the brain over time can make individuals more susceptible to the conditions that cause it (Mecocci and Boccardi, 2021). As populations continue to age, the prevalence of dementia is projected to rise, magnifying its impact on society, economies, families, and individuals. The main underlying causes of dementia include Alzheimer's disease (AD), vascular dementia, Lewy body dementia and frontotemporal dementia. AD, the most common underlying cause of dementia, accounts for 60-70% of dementia cases (World Health Organization, 2023). Dementia has historically been diagnosed according to the manifestation of clinical symptoms as measured through neuropsychological assessment, however, when it comes to AD, the field has increasingly adopted a biomarker-based classification, relying on evidence of specific pathological hallmarks such as amyloid plaques and tau neurofibrillary tangles (Jack et al., 2024).

Mild cognitive impairment (MCI), a clinical stage between normal aging and dementia, is often characterized by subtle cognitive changes that are noticeable yet not severe enough to significantly impede daily life (Petersen, 2004). Consequently, accurate MCI diagnosis has become a key focus for researchers, as it offers a critical window for early intervention before dementia fully manifests. This diagnostic precision is now even more vital with the advent of disease-modifying treatments that show efficacy in the early stages of Alzheimer's disease (Reardon, 2023). For instance, the drug Donanemab was found to reduce AD disease progression by 60% and cleared nearly 90% of brain amyloid, but only in patients at the early stages of AD (Reardon, 2023). Numerous studies have established a strong link between MCI and dementia, indicating that 10–15% of individuals with MCI develop Alzheimer-type dementia within 1 year, up to 50% progress within 3 years, and ~80% convert within 5 years (Amieva et al., 2004; Gauthier et al., 2006; Petersen et al., 1999). Additionally, other dementia pathologies, including vascular dementia, frontotemporal dementia, and Lewy body dementia, exhibit a 50% conversion rate from MCI to dementia within an average three-year period (Michaud et al., 2017). This provides an opportunity for timely interventions that may stop/slow progression through lifestyle interventions or drug therapies if MCI can be detected accurately.

The diagnosis of mild cognitive impairment (MCI) includes various subtypes that have been found to have varying correlations with different dementia pathologies (Petersen, 2016). MCI is commonly categorized into amnestic MCI (aMCI) and non-amnestic MCI (naMCI), based on whether memory impairment is present (Petersen, 2016). Both aMCI and naMCI can further be classified into single-domain or multi-domain MCI, depending on the number of cognitive domains affected (Michaud et al., 2017; Petersen, 2016). All MCI subtypes are more strongly linked to the progression of Alzheimer's disease (AD) compared to other forms of dementia, such as frontotemporal, vascular, or Lewy body dementia (Elkasaby et al., 2023). However, the strength of this relationship varies across subtypes. For example, aMCI and amnestic multi-domain MCI are more closely associated with the development of AD than other MCI subtypes (Elkasaby et al., 2023). This has led to a focus in research on amnestic subtypes for the early diagnosis of AD.

The general consensus on the clinical criteria for MCI involves a self- or informant-reported cognitive complaint alongside objective cognitive impairment measured through tests. Crucially, individuals with MCI maintain preserved independence in functional abilities, and their general cognitive functioning is essentially preserved, ensuring the criteria for no dementia is met (Petersen et al., 2014). Within the framework of MCI as a general clinical entity, the gold standard for diagnosis has therefore typically been comprehensive neuropsychological assessment. However, when focusing on the relationship between MCI and AD, recent revisions by the National Institute of Aging-Alzheimer's Association (NIA-AA) have fundamentally reoriented the diagnostic understanding of MCI due to AD, moving from a purely symptom-based approach to a biologically driven definition (Jack et al., 2024). This shift means that the NIA-AA's gold standard for research into MCI relies on a reference standard confirming the presence of amyloid-beta deposition and/or tau pathology in the brain, typically ascertained through methods such as amyloid PET imaging or cerebrospinal fluid (CSF) analysis (Jack et al., 2024).

However, these methods are often invasive, expensive, or time-intensive, making them impractical for routine or large-scale screening for AD (Wimo et al., 2024). For example, CSF testing requires lumbar punctures, which can be uncomfortable for patients, while MRI and PET scans involve lengthy procedures and significant costs, limiting their accessibility (Wimo et al., 2024). Eligibility for disease modifying therapies requires positive biomarkers as obtained through these methods (Belder et al., 2023). Nevertheless, as acknowledged by the NIA-AA, an initial screening process using more cost-effective methods is essential to determine patient eligibility for subsequent Alzheimer's disease biomarker assessment (Jack et al., 2024). Initial screening for cognitive impairment typically involves neuropsychological testing. Although comprehensive assessments are accurate, they are time-consuming. Conversely, rapid tools like the Mini-Mental State Examination (MMSE) and Montreal Cognitive Assessment (MoCA) are quick and non-invasive but often lack the sensitivity and specificity to detect early or subtle cognitive decline (Carson et al., 2018; Tsoi et al., 2015). Addressing the need for accessible and efficient pre-biomarker screening, current advancements in virtual reality (VR) technologies for neuropsychological assessment present a promising avenue for exploration.

In the research literature, the definition of VR varies significantly, with no clear consensus on what constitutes VR technology (Abbas et al., 2023). Traditionally, VR is associated with wearable headsets or goggles that immerse users in a fully 3D computer-generated environment. However, the term is also used more broadly to describe computer-generated simulations that replicate real places or situations, enabling users to interact in ways that feel realistic (Abbas et al., 2023). Under this broader definition, environments displayed on computer screens or tablet devices also qualify as VR, provided they allow users to engage with a simulated environment that appears authentic. Consequently, the research literature encompasses a wide range of paradigms under the umbrella of “VR research”. To address this variability, it has become essential to categorize VR paradigms based on their level of immersion (see Table 1).

**Table 1:** Different types of virtual reality definitions according to immersion degree (based on Liu et al., 2023).

**Feature**	**Immersive VR**	**Semi-immersive VR**	**Non-immersive VR**
Equipment	Head-mounted displays (VR-headsets),	Large screens, projection systems	Monitors or tablets.
User Experience	High immersion, fully isolates users in a virtual environment	Moderate immersion, partial presence	Fully aware of real-world environment while also aware of virtual environment
Interaction	Through head and body movements, often handheld controllers tracking movement	Physical controls or limited movement tracking	Indirect (via input devices, mouse and keyboard or touch)

VR technology is emerging as a highly promising tool for dementia screening, especially with advancements in machine-learning methods. VR assessments could generate large volumes of data by capturing detailed information on users' behaviors, movements, and responses in real-world-like scenarios. Traditionally, processing and interpreting such complex datasets posed significant challenges. However, modern machine-learning techniques now make it possible to analyze these data efficiently, possibly uncovering subtle and multidimensional patterns indicative of MCI that would otherwise go unnoticed. As an example, emerging evidence suggests that early dementia symptoms often manifest subtly in daily activities (Jekel et al., 2015), movement (Chen et al., 2020), eye movement (Opwonya et al., 2022), altered EEG patterns (Yang et al., 2019), and speech changes (Sanborn et al., 2022). Technology now exists to automatically collect modalities such as eye movement, bodily movements, speech, and EEG within a VR setup. Speech analysis requires only a microphone and appropriate machine-learning software. Low-cost, validated EEG devices in the form of wearable headbands are now widely available to the public and have been used in machine-learning studies to accurately detect MCI (Wu et al., 2023; Xue et al., 2023). Eye-tracking is often an integrated function of VR headsets, making implementation straightforward, while movement data can be captured using kinematic sensors or handheld controllers. Since these technologies rely on specific stimuli or tasks to elicit measurable responses, VR assessments may be uniquely positioned to integrate these technologies in dementia screening.

Unlike traditional tests, VR environments can simulate real-world situations, allowing researchers to measure how a patient's cognitive decline impacts their ability to navigate and interact with their surroundings. This approach may provide a direct way to study structure-function relationships, connecting specific changes in brain structure to observable declines in function. By doing so, VR may help us move beyond simple observation and could provide a more objective, measurable way to track the progression of dementia, potentially leading to earlier and more accurate diagnoses. As an example, studies suggest that the entorhinal cortex (EC) is fundamentally involved in navigation, thanks to its spatially-modulated neurons (Igarashi, 2023). As this is one of the first brain regions to show damage in Alzheimer's disease, a decline in its function could serve as an important biomarker for detecting the disease at its earliest onset. For instance, VR can be utilized to design tasks that assess EC-related cognitive functions. Studies by Howett et al. (2019) and Castegnaro et al. (2022) have implemented this by measuring participants' ability to retrace a path and recall object locations within a VR environment. Furthermore, the possibility of self-administered virtual assessments might also reduce the need for specialized personnel, increasing accessibility for at-risk populations. However, questions remain regarding the overall accuracy of virtual assessments, the potential enhancements offered by machine learning, optimal design strategies, and the most promising assistive technologies. Additionally, concerns remain about the feasibility of VR-assessments, including cost-effectiveness, time efficiency, acceptance among older populations, and whether VR assessments are better suited as screening tools for dementia compared to traditional methods.

The aim of our study is to explore the accuracy of current VR-based cognitive tests in differentiating patients with MCI from healthy controls through a meta-analysis and systematic review of relevant studies. Furthermore, we aim to provide an informative discussion on the feasibility of VR-based dementia screening and how advanced technologies and machine-learning may enhance dementia screening based on the findings of the included studies.

## 2 Method

A systematic review and meta-analysis were selected as the methodological approach to ensure a comprehensive, accurate, and transparent synthesis of the available evidence. This study was conducted in accordance with PRISMA 2020 guidelines (Page et al., 2021) to maintain methodological rigor. As outlined in section 2.4, the quality assessment of the included studies follows the QUADAS-2 framework (Whiting, 2011). The subsequent sections will describe the search strategy, inclusion and exclusion criteria, data extraction process, risk of bias assessment, and statistical methods employed.

### 2.1 Search strategy

The literature search was conducted from June 25 to September 17, 2024, using the PubMed, IEEE Xplore, and PsycINFO databases. The following search string was put together: (“virtual reality” OR “serious game” OR “virtual game” OR “video game” OR “augmented reality”) AND (“cognitive impairment” OR “mild cognitive impairment” OR “pre-dementia” OR “pre-alzheimer” OR “MCI”) AND (“screen^*^” OR “detect^*^” OR “predict^*^” OR “evaluate^*^” OR “diagnosis^*^” OR “assess^*^” OR “discriminate^*^” OR “machine learning” OR “deep learning” OR “artificial intelligence”). Citation searches were also performed in relevant review articles and eligible studies.

### 2.2 Inclusion and exclusion criteria

Eligibility for inclusion in the systematic review and meta-analysis was evaluated using the PICO model, as recommended by the Cochrane collaboration (Thomas et al., 2023). Table 2 summarizes the main PICO inclusion criteria used. In addition to meeting the PICO criteria, studies were required to be peer-reviewed and published in English. Articles that were preprints, guidelines, or review articles were excluded.

**Table 2:** PICO inclusion criteria.

Population	Participants diagnosed with MCI or MCI-subtypes according to established criteria
Intervention	Assessments using tools that are consistent with the broader definition of VR
Comparison	Healthy controls
Outcomes	Sensitivity and specificity or data that these measures can be derived from

Studies were included if they met the following criteria:

1. Studies must involve patients diagnosed with Mild Cognitive Impairment (MCI), including its subgroups (e.g., amnestic MCI), based on recognized diagnostic criteria e.g., the Petersen criteria or recommendations of the National Institute on Aging (Albert et al., 2011; Petersen, 2004).
2. Studies must use assessment tools that align with the broader definition of Virtual Reality (VR), meaning computerized simulations that replicate real places or situations and enable users to interact in ways that feel realistic.
3. Studies must report accuracy measures for differentiating MCI from healthy controls. For studies including multiple groups (MCI, healthy controls, and dementia), specific accuracy data for MCI vs. healthy controls must be provided.
4. Studies must provide data that allow for the calculation of diagnostic accuracy metrics (i.e., true positives, false positives, true negatives, false negatives, sensitivity, and specificity) or report these measures directly.

Studies were excluded if they met the following criteria:

1. Studies that include patient groups with already developed dementia without a specific focus on detecting MCI.
2. Studies that use computerized tests that do not replicate real-life situations or environments.
3. Studies that only report accuracy data for distinguishing MCI from dementia, without providing specific measures for differentiating MCI from healthy controls.
4. Studies that do not provide the necessary data (e.g., sensitivity, specificity, and participant numbers in the diagnostic groups) to compute or derive key diagnostic accuracy metrics.

### 2.3 Data extraction

Data from the relevant studies were organized into a data extraction table. The extracted information included the year of publication, author names, study location, type of assistive technologies used, neuropsychological tests administered, comparative tests, reference standards, and time to test completion. Additionally, studies were categorized by immersion degree (non-immersive, semi-immersive, or fully immersive) based on the definitions provided in the introduction (section 1.0). For the meta-analysis, diagnostic accuracy data such as specificity, sensitivity, true positives, false positives, true negatives, and false negatives were compiled into tables for statistical analysis. Diagnostic accuracy studies often report performance across various cut-off values. When multiple cut-off values were provided, the cut-off recommended by the authors of the included study was used for the meta-analysis. If no recommendation was available, the cut-off highlighted in the abstract of the included study was selected. In cases where multiple machine-learning models were compared and no recommendation was provided, data from the model with the highest average of specificity and sensitivity were included in the meta-analysis.

### 2.4 Risk of bias and study quality assessment

To ensure quality and evaluate risk of bias in the different studies the Quality Assessment of Diagnostic Accuracy Studies 2 instrument (QUADAS-2) was used (Whiting, 2011). The QUADAS- 2 focuses on four key domains to assess the reliability of the study's results. First, it examines patient selection, determining whether the inclusion of participants was free from bias, particularly avoiding inappropriate exclusions that could skew the results and whether a case-control design was avoided. Second, it assesses the index test, looking at how the test being evaluated (e.g., new screening test or diagnostic tool) was conducted and whether its results were interpreted consistently and in a pre-specified manner. Third, an evaluation of the risk of bias in the reference standard, which is the diagnostic method used as a benchmark to assess the accuracy of the index test (test being developed). This domain checks whether the reference standard is appropriate and applied consistently throughout the study and in line with the current gold standard for diagnosis. As the gold standard for diagnosing mild cognitive impairment, a multimodal approach combining neuropsychological testing, clinical judgment, and functional assessments is typically recommended. Following widely accepted criteria like the NIA-AA or Petersen criteria ensures that the diagnosis is robust and can be compared across studies (Albert et al., 2013; Petersen, 2004). Lastly, QUADAS-2 examines flow and timing, ensuring that there is a reasonable time interval between the application of the index test and the reference standard, and that no participants were excluded after the study started without proper explanation. The risk of bias will be categorized as low, unclear, moderate, or high based on the QUADAS-2 domains.

However, as machine-learning models are being used in a good portion of the included studies, this brings a new dimension to the quality assessment process. The QUADAS-2 index domain will therefore be switched out with an assessment of the machine-learning validation method being employed. Currently a new edition of the QUADAS-2 for studies using AI is being developed (QUADAS-AI), but has not yet been published and is expected to be finished by late 2024 to early 2025 (Guni et al., 2024). The main differences will probably be in the index test assessment section, as machine-learning is being used in the index test. Based on the current literature, machine-learning validation methods using cross-validation will be assigned “low-risk”, methods using the holdout-method will be assigned “moderate-risk” and finally methods that do not split the data-set into training and test (resubstitution method) will be assigned “high-risk” (see Table 3). It is important to note that the risk of bias in the holdout method is also influenced by sample size. When sample sizes are large, the risk of bias is minimal. This will be accounted for when evaluating risk of bias. In summary, the QUADAS-2 domains are used for assessing risk of bias in the included machine-learning studies, with the index domain adjusted to evaluate risk of bias according to the validation method being used. There are several types of validation methods, but a more in-depth explanation is outside the scope of this article. For a deeper explanation of machine-learning validation methods see Diniz (2022).

**Table 3:** Machine-learning validation methods and associated risk of bias.

**Validation method**	**Risk level**	**Explanation**
Cross-validation	Low risk	Uses multiple data splits to evaluate the model, reducing overfitting and variance across different folds.
Holdout method	Medium risk	Involves a single data split, which can lead to biased results due to dependence on one specific data division. (Does not introduce great bias if samples are large).
Resubstitution method	High risk	Evaluates the model on the same data used for training, leading to overfitting and unrealistic performance metrics.

### 2.5 Statistical analysis

All statistical analyses and associated figures were generated using MetaBayesDTA, a Bayesian hierarchical model specifically designed for meta-analyses of diagnostic test accuracy (Cerullo et al., 2023; Freeman et al., 2019; Patel et al., 2021). This approach provides robust estimates by incorporating prior information and accounting for uncertainty. Given the limited prior knowledge about the sensitivity, specificity, and heterogeneity of VR-based assessments, weakly informative priors were employed to allow the results to be driven primarily by the data.

The priors used in the model were as follows:

- Logit sensitivities and specificities: normal distribution with a mean of 0 and SD of 1 (95% prior interval: 0.05–0.95 on the probability scale).
- Between-study standard deviations: truncated normal distribution with a mean of 0 and SD of 1, truncated at 0 (95% prior interval: 0.03–2.25).
- Between-study correlation between sensitivities and specificities: LKJ(2) prior (95% prior interval:−0.8 to 0.8).

A pooled estimate of sensitivity, specificity, and overall diagnostic accuracy was calculated using a bivariate random-effects model in MetaBayesDTA. This model, widely regarded as the standard for diagnostic test accuracy meta-analysis (Reitsma et al., 2005), accounts for variability both within studies (due to sampling error) and between studies (due to differences in design, populations, or thresholds). This ensures that both within- and between-study variability are appropriately managed.

The pooled estimates are reported with 95% credible intervals, which represent the Bayesian equivalent of confidence intervals, indicating the range where the true diagnostic performance is likely to lie with 95% certainty. Forest plots were generated to display sensitivity and specificity estimates from each study alongside their respective credible intervals, providing a clear visual summary of the variation across studies. Additionally, a Hierarchical Summary Receiver Operating Characteristic (HSROC) curve was produced to illustrate the balance between sensitivity and specificity across the included studies, offering an overarching view of the overall diagnostic performance. Furthermore, subgroup and sensitivity analyses were conducted. Publication bias was examined using Deeks' funnel plot, with the statistical significance assessed via Deeks' asymmetry test in R.

## 3 Results

The following section presents the results of the literature search, followed by characteristics of the included studies, including immersion degree, technologies used, MCI subtypes, test types, and countries of origin. Additionally, the results from the statistical analysis and quality assessment of the included studies are provided. Since approximately half of the studies employed machine-learning methods for diagnosis, the risk of bias analysis is divided into two categories: studies using machine learning and those that do not. This division is appropriate due to the significant differences between machine-learning-based methods and conventional approaches. Furthermore, a comparison of the screening performance of machine-learning-enhanced methods vs. non-machine-learning methods is provided.

### 3.1 Literature search

**Figure 1:**
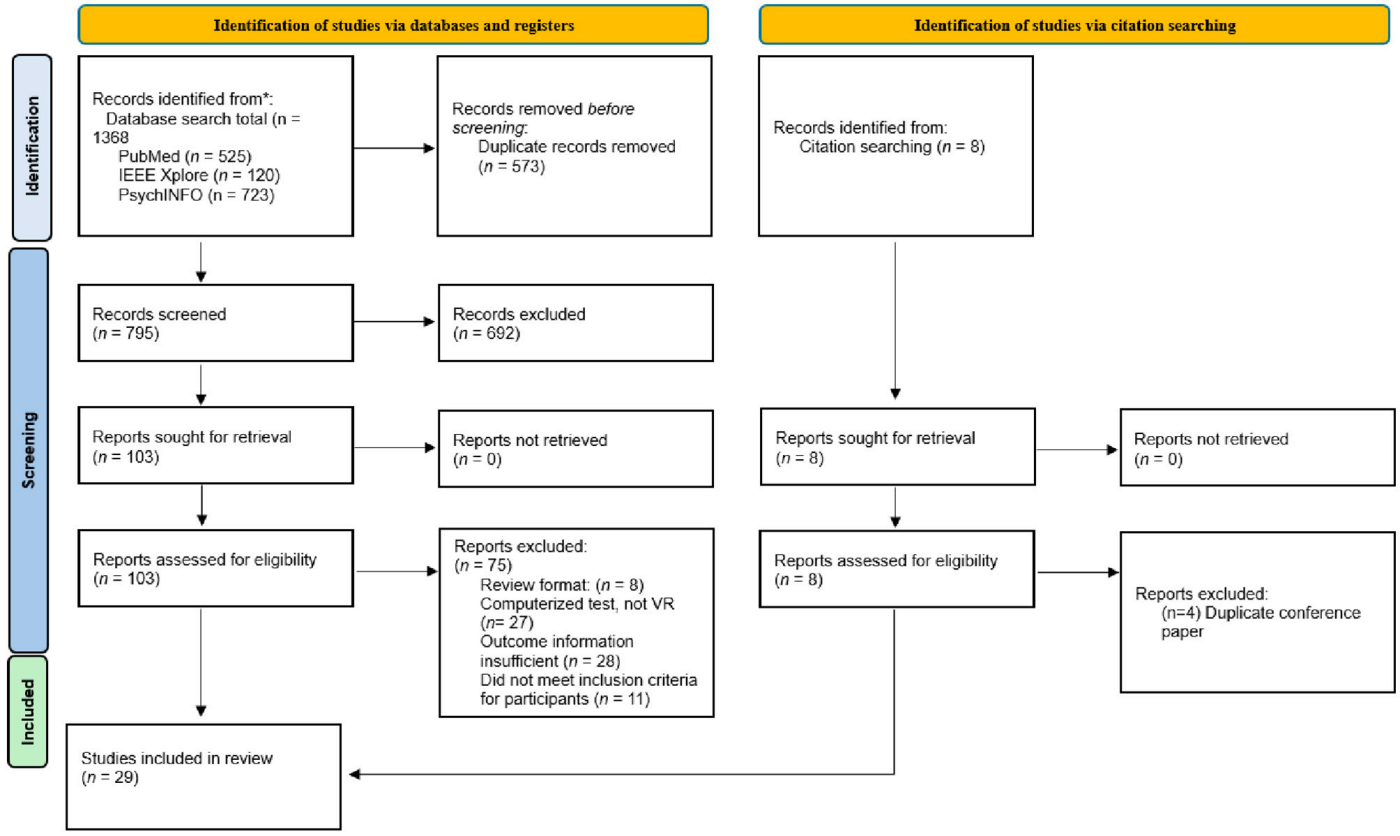
PRISMA flow-chart.

Figure 1 presents a PRISMA flowchart illustrating the study selection process. A total of 1,368 articles were identified through database searches in PubMed (*n* = 525), IEEE Xplore (*n* = 120), and PsycINFO (*n* = 723), with an additional 8 articles identified through citation searching. After removing 573 duplicate articles, 795 articles remained for title screening. During title screening, 692 articles were excluded, primarily due to their focus on diagnoses unrelated to MCI, such as schizophrenia, phobias, PTSD, ADHD, neglect, or executive dysfunction. Additionally, several excluded articles were reviews, explored VR technology in rehabilitation rather than screening or diagnosis, or did not involve any form of virtual reality technology. Following title screening, 103 articles were selected for full-text review. From the full-text review, 75 articles were excluded for the following reasons: being review articles (*n* = 8), not meeting the definition of VR (*n* = 27), lacking sufficient outcome information (*n* = 28), or including participants outside the inclusion criteria (*n* = 11). Additionally, 4 articles identified through citation searching were excluded as they were conference papers duplicating existing studies. This process resulted in 29 articles being included in the final review.

### 3.2 Characteristics of the included studies

The final literature review and meta-analysis included 29 studies (see Table 4), 13 of which employed machine learning as an assistive technology. The studies were published between 2012 and 2024, with 19 appearing after 2020, reflecting the rapid growth of VR-related research in recent years. This trend is particularly pronounced for VR-assisted screening using machine learning: all but one of the 13 machine learning studies were published after 2020, with 9 published since 2023 alone. Collectively, the machine-learning-based studies included 1,366 participants, while the non-machine-learning studies involved 1,389 participants. The research spanned a diverse range of countries, with the highest representation from South Korea (*n* = 10), followed by Greece (*n* = 5), China (*n* = 4), Italy (*n* = 3), and one study each from Turkey, Singapore, Brazil, Saudi Arabia, Taiwan, Spain, and the United States. The mean age of participants ranged from 62.3 to 77.7 years, with most studies reporting a mean age above 70.

**Table 4:** Characteristics of the included VR-assessments.

**Author**	**Immersion degree**	**Technologies**	**Screening test**	**Participants**	**Participants' age**	**Country**	**Cognitive domains assessed**	**Diagnostic MCI criteria used**	**Time to administer**	**Accuracy metrics**
Kim D. et al. (2024)	Full	EEG-SSVEP, Eye-tracking, movement tracking Machine-learning	Virtual kiosk test	24 aMCI 24 HC	Mean age: 70	South-Korea	Executive functioning, Visuospatial processing, Processing speed	NIA-AA 2013. (Albert et al., 2013)	5–15 min	Sensitivity: 0.958 Specificity: 1.0
Park et al. (2024)	Full	Eye- tracking, movement-tracking MRI-data Machine-learning	Virtual kiosk test	32 MCI 22 HC	Mean age: 71.7	South-Korea	Executive functioning, Visuospatial processing, Processing speed	NIA-AA 2011 criteria (Albert et al., 2011)	5–15 min	Sensitivity: 0.875 Specificity: 0.909
Kallel et al. (2024)	Full	Eye-tracking, Movement tracking, EEG-data MRI-data Machine-learning	Virtual kiosk test	32MCI 22 HC	N/A	South-Korea	Executive functioning, Visuospatial processing, Processing speed	Unspecified	5–15 min	Sensitivity: 0.727 Specificity: 0.867
Kim S. Y. et al. (2024)	Full	Eye- tracking, Movement tracking, EEG, MRI-data, Machine-learning	Virtual kiosk test	25 MCI 21 HC	N/A	South-Korea	Executive functioning, Visuospatial processing, Processing speed	NIA-AA 2011 criteria (Albert et al., 2011)	5–15 min	Sensitivity: 1.0 Specificity: 0.762
Xu et al. (2024)	Full	Eye-tracking, Machine-learning	VECA	MCI 60 HC 141	55–65: 113 65–75: 79 75+: 9	China	Executive functioning, Visuospatial processing, Processing speed	Unspecified Only used MoCA	5 min	Sensitivity: 0.885 Specificity: 0.830
Kim et al. (2023)	Full	Eye-tracking, Movement-tracking Machine-learning	Virtual kiosk test	31 MCI 20 HC	Mean age: 71.82	South-Korea	Executive functioning, Visuospatial processing, Processing speed	NIA-AA 2011 criteria (Albert et al., 2011)	5–15 min	Sensitivity: 1.000 Specificity: 0.850
Wu et al. (2023)	Full	EEG, Speech analysis Machine-learning	N/A	44 MCI 42 HC	Mean age: 68.2	China	Language Executive functioning, Visuospatial processing	Unspecified MMSE+MoCA	Unspecified	Sensitivity: 0.886 Specificity: 0.905
Xue et al. (2023)	Full	EEG Machine-learning	VRNPT	40 MCI 40 HC	Mean age: 62.3	China	Attention, Visuospatial processing, Working memory	NIA-AA 2011 criteria (Albert et al., 2011)	17 min	Sensitivity: 0.900 Specificity: 0.875
Bayahya et al. (2022)	Full	Machine-learning	MVD	30 MCI 60 HC	N/A	Saudi Arabia	Spatial navigation, Visuospatial processing, Long-term memory (delayed recall)	Unspecified	Unspecified	Sensitivity: 0.850 Specificity: 1.00
Lee et al. (2022)	Full	EEG Machine-learning	N/A	21 MCI 22 HC	Mean age: 70.4	South Korea	Attention, Working memory, Visuospatial processing	Petersen criteria (Petersen and Morris, 2005)	Less than 30 min	Sensitivity: 0.731 Specificity: 0.821
Tsai et al. (2021)	Full	Machine-learning	Virtual supermarket	6 MCI 6 HC	Mean age: 72.4	Taiwan	Long term memory (delayed recall) Spatial navigation, Executive functioning	Unspecified	Unspecified	Sensitivity: 1.0 Specificity: 1.0
Buegler et al. (2020)	Semi	Movement analysis, Augmented reality, Machine-learning	Altoida digital neuro signature (DNS)	213 MCI 283 HC	Mean age: 67	Mulitcenter; USA + 7 European countries	Executive functioning, Spatial memory/navigation, Processing speed	NIA-AA 2011 criteria (Jack et al., 2011) Used AD Biomarkers	10 min	Sensitivity: 0.840 Specificity: 0.880
Valladares-Rodriguez et al. (2018)	Low	Machine-learning	Episodix	28 HC 16 MCI	Mean age: 77.03	Spain	Working memory, Short-term memory, Long-term memory (delayed recall)	NIA-AA 2011, Petersen criteria (Petersen, 2004; Albert et al., 2011)	Unspecified	Sensitivity: 0.938 Specificity: 0.857
Zygouris et al. (2015)	Low	None	Virtual supermarket test (VST)	34 MCI 21 HC	Mean: 68.9	Greece	Executive functioning, Short-term memory, Spatial navigation	Petersen criteria (Petersen, 2004)	Unspecified	Sensitivity: 0.824 Specificity: 0.952
Zygouris et al. (2017)	Low	None	VST	6 MCI 6 HC	Mean age: 63.7	Greece	Executive functioning, Short-term memory, Spatial navigation	Petersen 2004 criteria (Petersen, 2004)	Unspecified	Sensitivity: 1.0 Specificity: 0.833
Eraslan Boz et al. (2019)	Low	None	VST	37 aMCI 52 HC	Mean age: 69	Turkey	Executive functioning, Short-term memory, Spatial navigation	Petersen criteria, (Petersen et al., 2009)	25 min	Sensitivity: 0.784 Specificity: 0.865
Zygouris et al. (2020)	Low	None	VST	47 MCI 48 HC	Mean age: 66.9	Greece	Executive functioning, Short-term memory, Spatial navigation	Petersen criteria, (Petersen et al., 2009)	30 min	Sensitivity: 0.766 Specificity: 0.917
Yan et al. (2021)	Low	None	Virtual supermarket program	62 MCI 64 HC	Mean age: 77.7	China	Executive functions, Short term memory, Spatial navigation	Petersen criteria, (Petersen, 2004)	Unspecified	Sensitivity: 0.857 Specificity 0.797
Cabinio et al. (2020)	Low	None	Smart aging serious game (SASG)	32 aMCI 107 HC	Mean age: 76.5	Italy	Executive functioning, Working memory, Attention, Visuospatial processing, Long-term memory (delayed recall)	DSM-V criteria, NIA-AA criteia 2011 [American Psychiatric Association (APA), 2013; Albert et al., 2011] Used AD Biomarkers	Unspecified	Sensitivity: 0.833 Specificity: 0.757
Isernia et al. (2021)	Low	None	SASG	87 MCI 74 HC	Mean age: 74.6	Italy	Executive functioning, Working memory, Attention, Visuospatial processing, Long-term memory (delayed recall)	NIA-AA 2011 criteria (Albert et al., 2011) Used AD Biomarkers	Unspecified	Sensitivity: 0.767 Specificity: 0.730
Caffò et al. (2012)	Low	None	Virtual Reorientation Test	51 aMCI 53 HC	Mean age: 70.5	Italy	Reorientation, Spatial navigation	Petersen criteria (Petersen, 2004)	Unspecified	Sensitivity: 0.804 Specificity 0.943
Chua et al. (2019)	Semi	None	REACH assessment module	23 MCI 37 HC	Mean age: 71.9	Singapore	Executive functioning, Visuospatial processing, Working memory	Unspecified Only used the MoCA	19–20 min	Sensitivity: 0.783 Specificity: 0.757
Jang et al. (2023)	Full	None	VARABOM Test	12 MCI 108 HC	Mean age: 74	South Korea	Executive functioning, Working memory, Visuospatial processing, Attention	Unspecified Only used the general dementia scale (GDS)	19 min	Sensitivity: 0.833 Specificity: 0.722
Da Costa et al. (2021)	Low	None	SOIVET Maze Task	MCI 19 HC 29	Mean age: 71.3	Brazil	Spatial navigation, Working memory	Petersen criteria (Petersen, 2004)	Unspecified	Sensitivity: 0.737 Specificity 0.621
Seo et al. (2017)	Full	Motion tracking	Virtual daily living test (VDLT)	20 MCI 22 HC	Mean age: 72.4	South Korea	Executive functioning, Short-term memory	NIA-AA 2011 criteria (Albert et al., 2011)	Unspecified	Sensitivity 0.900 Specificity: 0.909
Tarnanas et al. (2013)	Full	Motion tracking, dual-belt treadmill	Viritual day out (VR-DOT)	65 aMCI 72 HC	Mean age: 72.7	Greece	Executive functioning, Working memory (spatial memory)	Petersen criteria (Petersen, 2004)	Unspecified	Sensitivity: 0.969 Specificity 1.000
Tarnanas et al. (2015a)	Semi	None	VAP-M	25 aMCI 25 HC	Mean age: 64.3	Greece	Working memory (spatial memory), Executive functioning, Visuospatial processing	Petersen criteria, (Petersen, 2004)	30 min	Sensitivity: 1.000 Specificity: 0.960
Tarnanas et al. (2015b)	Full	Unclear	VR-DOT	61 MCI 71 HC	Mean age: 72.1	Greece	Executive functioning, Visuospatial processing, Prospective memory, Spatial navigation	International Working Group 2004 criteria (Winblad et al., 2004)	Unspecified	Sensitivity: 1.000 Specificity: 0.944
Park (2022)	Low	None	SCT-VR	36 MCI 56 HC	Mean age: 74	South Korea	Spatial navigation	Petersen 2004 criteria (Petersen, 2004)	Unspecified	Sensitivity: 0.944 Specificity: 0.929

MCI, mild cognitive impairment; HC, healthy control; aMCI, amnestic mild cognitive impairment; NIA-AA, National Institute on Aging-Alzheimer's Association; MMSE, mini-mental state exam; MoCA, Montreal cognitive assessment; DSM-V, diagnostic statistical manual-V.

Of the 29 studies, 6 specifically assessed the accuracy of VR-based assessments for patients with amnestic mild cognitive impairment (aMCI), while 23 focused on all MCI subtypes. In terms of immersion levels, 15 studies were fully immersive, utilizing VR headsets to create complete virtual environments. Three were semi-immersive, employing large screens or projections to provide partial immersion, while 8 were non-immersive, relying on devices like tablets or desktop computers. Most studies utilized cross-sectional designs, except for four (Buegler et al., 2020; Tarnanas et al., 2013; Zygouris et al., 2017; Tarnanas et al., 2015b), which employed longitudinal designs.

As shown in Table 4, machine-learning studies are most prevalent in Asian countries, with 11 out of 14 studies originating from this region, including seven from South Korea. The trend of increased machine-learning studies in countries like South Korea may be linked to the significant challenges posed by rapidly aging populations. South Korea is projected to become a “super-aged society” by 2025, with over 20% of its population aged 65 or older (Statistics Korea, 2022). Another key observation is the notable difference in the level of immersion in machine-learning studies compared to non-machine-learning studies. This difference likely reflects the more recent publication dates of machine-learning studies, coinciding with the growing accessibility and adoption of modern VR headsets.

#### 3.2.1 Common trends and patterns among VR assessments

To emphasize the common patterns and trends among the studies included in this review, the following section provides a summary of the tasks and procedures frequently used in these assessments. This overview is necessary to clarify what VR-based assessments typically involve, as this may not be immediately intuitive to the reader, while also highlighting key characteristics of the included studies.

**Figure 2:**
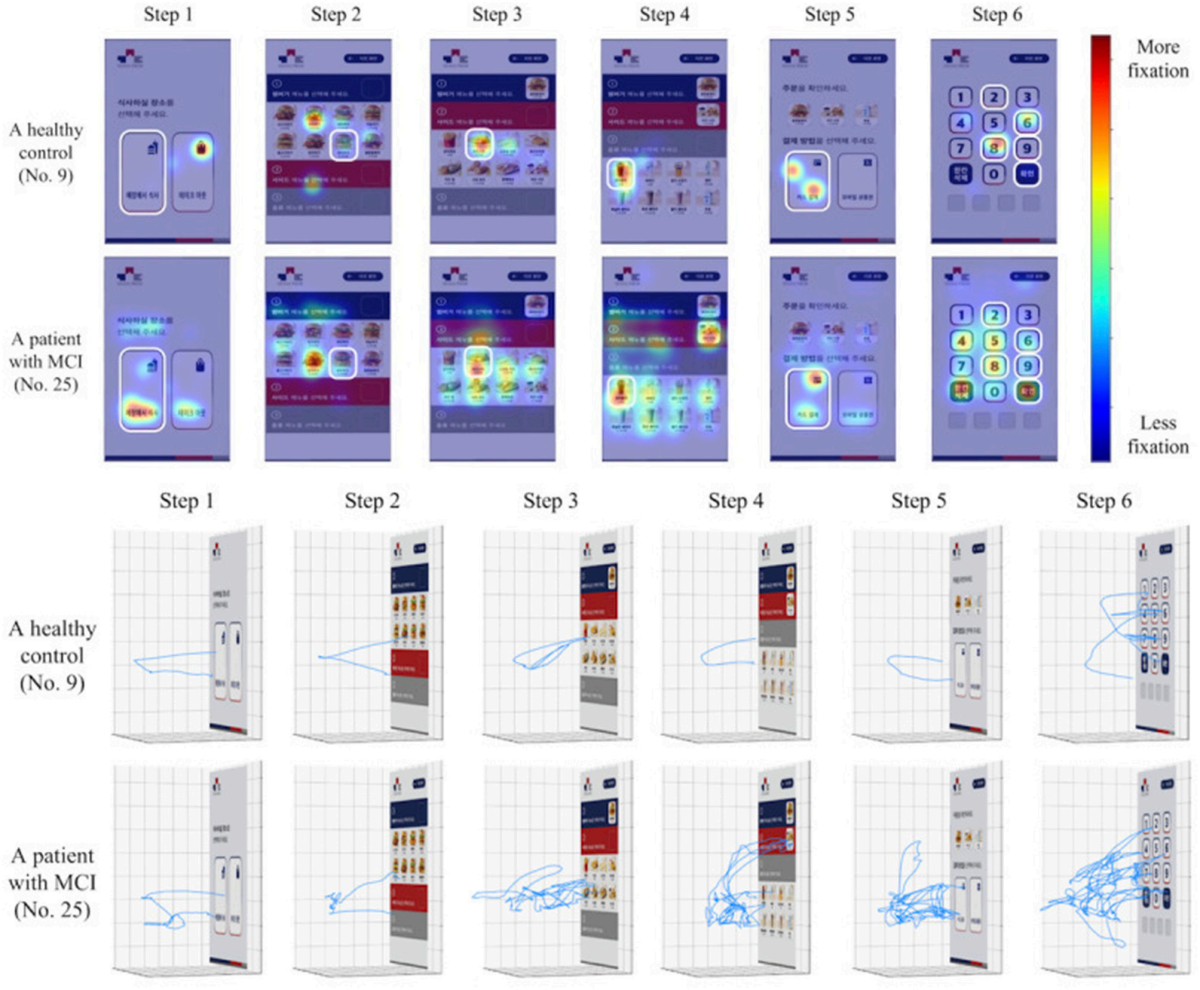
Comparison of a healthy control and an MCI patient's hand-movement and eye movement fixation patterns in the virtual kiosk test as illustrated in the original study by Kim et al. (2023).

Nine of the included studies (Kallel et al., 2024; Kim et al., 2023; Kim D. et al., 2024; Kim S. Y. et al., 2024; Park et al., 2024; Seo et al., 2017; Tarnanas et al., 2013; Buegler et al., 2020) utilized movement data as predictive variables for detecting MCI. Of the nine movement data studies, five studies (Kallel et al., 2024; Kim et al., 2023; Kim D. et al., 2024; Kim S. Y. et al., 2024; Park et al., 2024) used an immersive virtual test, called the virtual kiosk test, a test specifically designed to be used in conjunction with machine-learning, eye-movement data and hand movement data. Using a head-mounted display and hand controllers, participants complete a six-step task: choosing a dining location, selecting the instructed main course, side dish, and drink, choosing a payment method, and remembering a four-digit payment code (Kim et al., 2023). Throughout the task, behavioral data from hand and eye-movements are recorded. The collected data from these metrics are then used by a machine learning model to differentiate MCI from normal aging (Figure 2). Furthermore, of the virtual kiosk studies, three studies used the test in conjunction with an EEG recording device (Kallel et al., 2024; Kim D. et al., 2024; Kim S. Y. et al., 2024), and three studies, also fed the machine-learning model MRI-data (Park et al., 2024; Kim S. Y. et al., 2024; Kallel et al., 2024).

A total of six studies (Kallel et al., 2024; Kim D. et al., 2024; Kim S. Y. et al., 2024; Lee et al., 2022; Wu et al., 2023; Xue et al., 2023) used EEG-assisted tests, of which three (Lee et al., 2022; Wu et al., 2023; Xue et al., 2023) developed tests, specifically made for use in conjunction with EEG-recording. As an example, one of the included studies (Lee et al., 2022) used a test paradigm that integrates a wearable EEG device with a virtual reality head-mounted display and hand controllers. In the VR test, participants perform four tasks that measure different cognitive functions (Lee et al., 2022). During the VR tasks, EEG data is synchronized with task performance metrics, enabling a detailed analysis of how neural dynamics correspond to behavioral responses (Lee et al., 2022).

In one of the included studies, Wu et al. (2023) developed a test that integrates EEG data and speech analysis software. During the test, participants are presented with two virtual scenes and asked to provide detailed verbal descriptions. The system collects 49 speech features, which, along with EEG data, are subsequently processed using machine-learning algorithms for classification (Wu et al., 2023).

A key pattern observed across the studies is the use of various activities of daily living (ADL) in the VR-assessment. Only eight studies did not integrate ADL as a core component of their assessments (Bayahya et al., 2022; Caffò et al., 2012; Da Costa et al., 2021; Lee et al., 2022; Park, 2022; Valladares-Rodriguez et al., 2018; Wu et al., 2023; Xue et al., 2023). The ADL-based studies incorporated a range of practical tasks, such as completing a fire evacuation scenario (Tarnanas et al., 2013, 2015b), taking the bus and using an ATM (Seo et al., 2017), performing household chores (Cabinio et al., 2020; Chua et al., 2019; Isernia et al., 2021), searching for hidden items in a home environment (Buegler et al., 2020), visiting a museum (Tarnanas et al., 2015a), or caring for a grandchild (Jang et al., 2023).

Five of these ADL-studies (Eraslan Boz et al., 2019; Tsai et al., 2021; Zygouris et al., 2015, 2017, 2020) used tests in a virtual supermarket setting. As an example, in the Virtual Supermarket Test (VST), first developed by Zygouris et al. (2015), a shopping list appears on the screen, and the participant must find items, place them in a cart, go to the cashier, and pay correctly. The VST is scored on how many correct items are collected, if the right amount is collected, if wrong items are collected, time to completion, and if the correct amount of money is used to pay for the items at the cashier. The VST was translated for use in Turkey in 2019 (Eraslan Boz et al., 2019) and in their 2021 study, Yan et al. developed a similar, but different supermarket test: the virtual supermarket program (VSP), adjusted for Chinese cultural habits. Furthermore, a machine-learning assisted version was developed by Tsai et al. (2021).

### 3.3 Statistical results

The bivariate random effects model gave a pooled sensitivity of 0.883 (95% CI: 0.846-0.918) and 0.887 specificity (95% CI: 0.846-0.920) when analyzing all the included studies. This amounts to a pooled detection accuracy of 88.5% for the included VR-assessment studies. This analysis weighted studies according to study variance, sample balance, and the number of participants (see Table 5). Sensitivities ranged from 0.727 to 1.0, while specificities varied from 0.722 to 1.0. Among the 2,923 participants, 1,396 had an MCI diagnosis, and the VR assessments successfully identified 1,232 of them.

**Table 5:** Diagnostic accuracy, classification and weighting toward pooled sensitivity and specificity.

**Author**	**TP**	**FN**	**FP**	**TN**	**N**	**Sens**	**Spec**	**Weight_Sens**	**Weight_Spec**
Bayahya et al. (2022)	17	3	0	65	85	0.85	1.0	2.808%	2.904%
Eraslan Boz et al. (2019)	29	8	7	45	89	0.784	0.865	4.105%	4.159%
Buegler et al. (2020)	179	34	34	249	496	0.84	0.88	5.073%	5.305%
Cabinio et al. (2020)	30	6	26	81	143	0.833	0.757	4.044%	5.06%
Caffò et al. (2012)	41	10	3	50	104	0.804	0.943	4.299%	3.644%
Chua et al. (2019)	18	5	9	28	60	0.783	0.757	3.678%	4.177%
Da Costa et al. (2021)	14	5	11	18	48	0.737	0.621	3.625%	4.17%
Isernia et al. (2021)	46	14	20	54	134	0.767	0.73	4.607%	4.887%
Jang et al. (2023)	10	2	30	78	120	0.833	0.722	2.843%	5.079%
Kallel et al. (2024)	16	6	2	13	37	0.727	0.867	3.677%	2.672%
Kim et al. (2023)	31	0	3	17	51	1.0	0.85	2.916%	2.678%
Kim D. et al. (2024)	23	1	0	24	48	0.958	1.0	2.598%	1.978%
Kim S. Y. et al. (2024)	25	0	5	16	46	1.0	0.762	2.885%	3.201%
Lee et al. (2022)	19	7	5	23	54	0.731	0.821	3.888%	3.65%
Park (2022)	34	2	4	52	92	0.944	0.929	3.33%	3.614%
Park et al. (2024)	28	4	2	20	54	0.875	0.909	3.613%	2.746%
Seo et al. (2017)	18	2	2	20	42	0.9	0.909	2.936%	2.729%
Tarnanas et al. (2013)	63	2	0	72	137	0.969	1.0	3.404%	2.645%
Tarnanas et al. (2015a)	25	0	1	24	50	1.0	0.96	2.509%	2.227%
Tarnanas et al. (2015b)	61	0	4	67	132	1.0	0.944	3.21%	3.554%
Tsai et al. (2021)	6	0	0	6	12	1.0	1.0	1.286%	1.023%
Valladares-Rodriguez et al. (2018)	15	1	4	24	44	0.938	0.857	2.627%	3.286%
Wu et al. (2023)	39	5	4	38	86	0.886	0.905	3.894%	3.578%
Xu et al. (2024)	54	7	24	117	202	0.885	0.83	4.29%	5.076%
Xue et al. (2023)	36	4	5	35	80	0.9	0.875	3.782%	3.68%
Yan et al. (2021)	54	9	13	51	127	0.857	0.797	4.428%	4.594%
Zygouris et al. (2015)	28	6	1	20	55	0.824	0.952	3.846%	2.562%
Zygouris et al. (2017)	6	0	1	5	12	1.0	0.833	1.449%	1.327%
Zygouris et al. (2020)	36	11	4	44	95	0.766	0.917	4.351%	3.792%

TP, true positives; FN, false negatives; FP, false positives, TN, true negatives and percentage contribution to the pooled sensitivity and specificity [(Weight %)].

**Figure 3:**
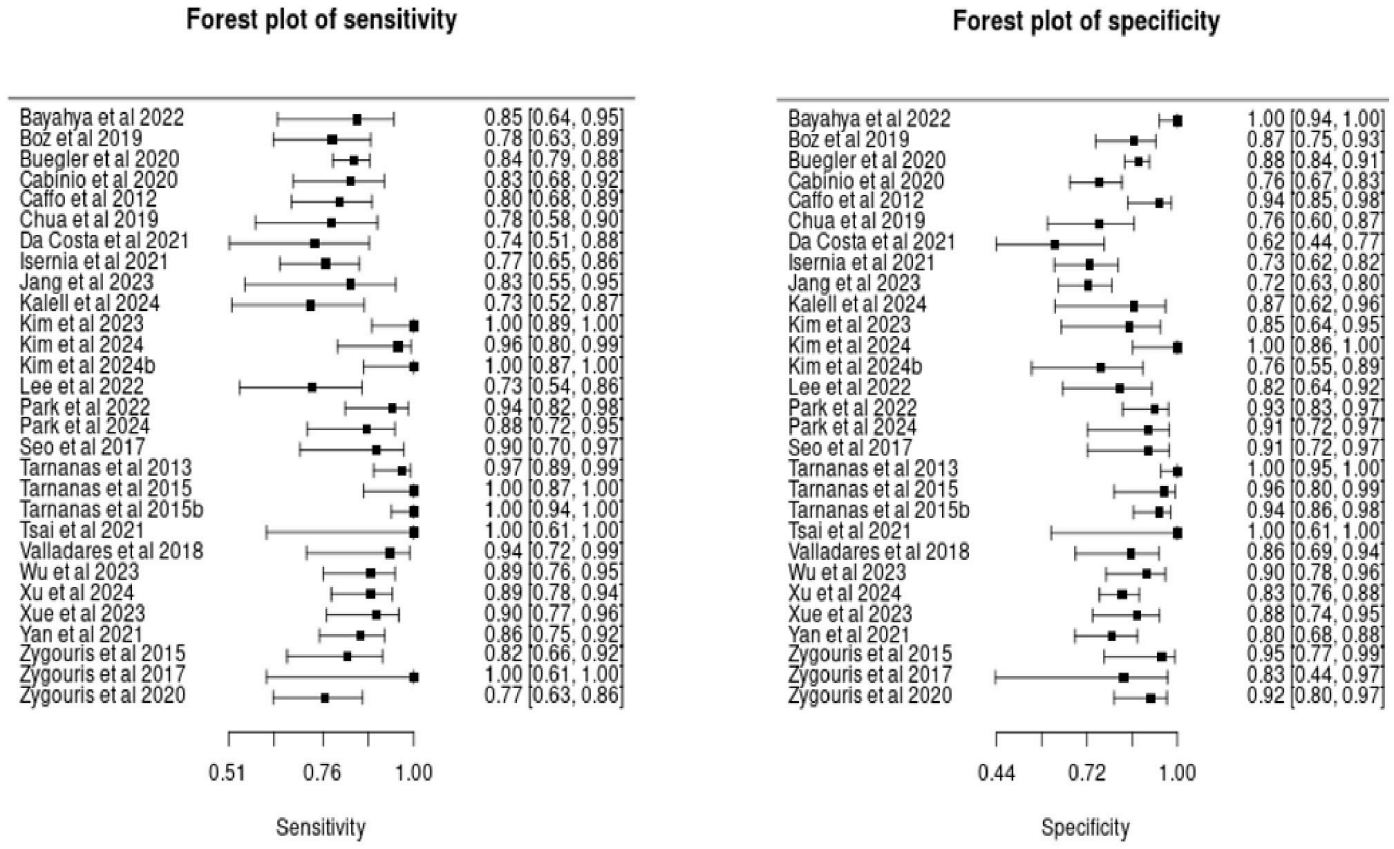
Forest plots of sensitivity and specificity estimates in included studies.

As illustrated in the forest plots (see Figure 3), many of the included studies exhibit uncertainty in their sensitivity and specificity estimates, reflected in the wide confidence intervals. This variability is largely due to the small sample sizes in several studies.

**Figure 4:**
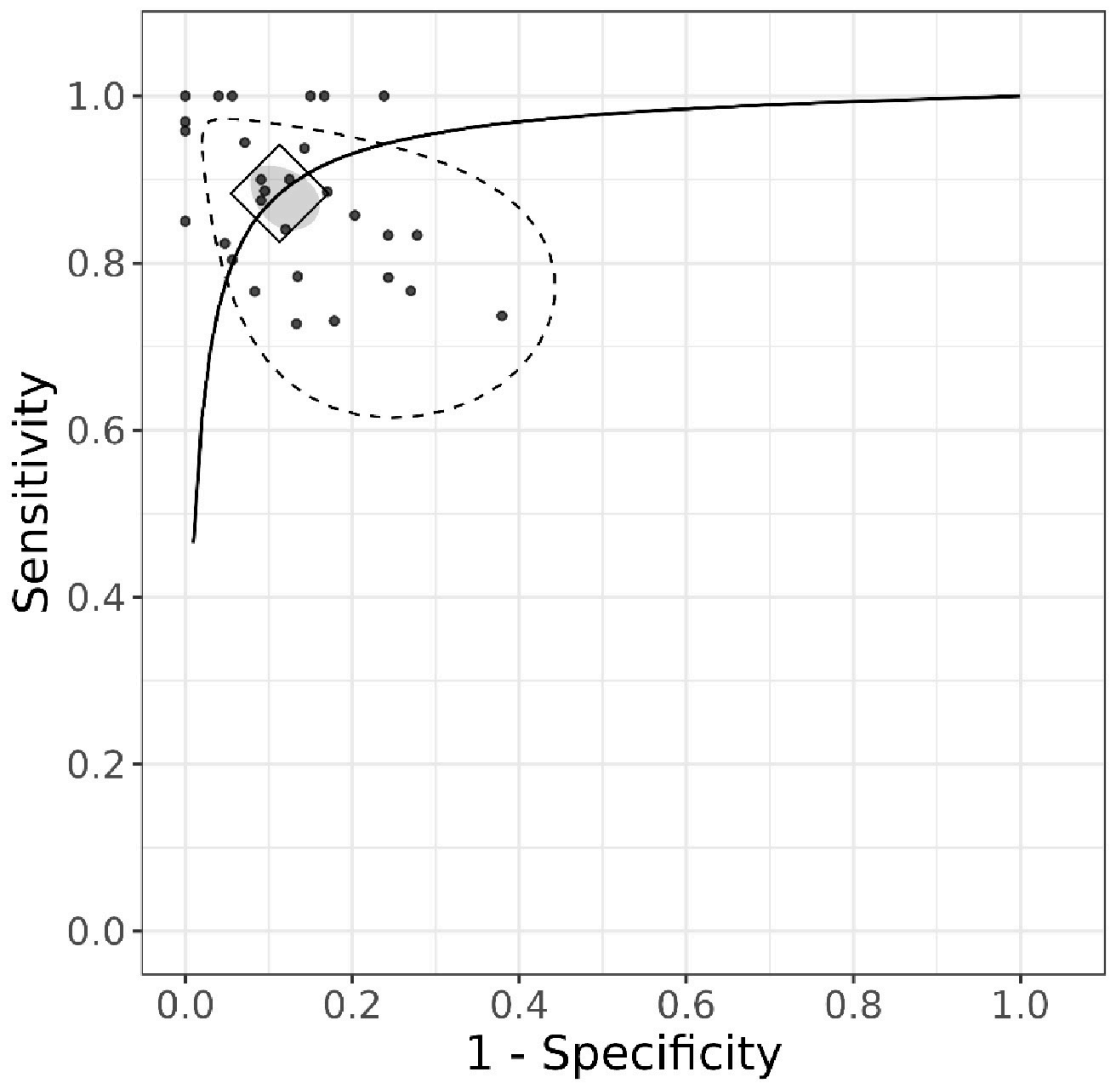
HSROC curve.

The hierarchical summary receiver operating characteristic (HSROC) curve (Figure 4) illustrates the diagnostic performance of the included studies. The diamond-shaped marker on the curve represents the pooled summary point, indicating the overall sensitivity and specificity estimated from the meta-analysis. The position of this summary point near the top-left corner of the plot suggests high diagnostic accuracy.

Additionally, the gray ellipse surrounding the summary point represents the 95% confidence region, highlighting the uncertainty around the pooled estimates. Unlike the credible intervals for individual studies in the forest plot, the confidence region around the pooled estimates is relatively small. This suggests that combining the studies in the analysis provides greater precision and higher certainty in the overall estimates. The stippled ellipse represents the 95% credible region, indicating where the model predicts future studies are likely to fall.

### 3.4 Sub-group analysis

When only analyzing studies that utilized machine learning, the 13 machine-learning-studies yielded a pooled sensitivity of 0.888 (95% CI: 0.845–0.930) and a specificity of 0.885 (95% CI: 0.842–0.929). This amounts to a pooled accuracy of 88.7% for machine-learning studies. Similarly, the 16 studies that did not incorporate machine learning showed a pooled sensitivity of 0.871 (95% CI: 0.796–0.924) and a specificity of 0.878 (95% CI: 0.804–0.931). This amounts to a pooled accuracy of 87.5%.

**Figure 5:**
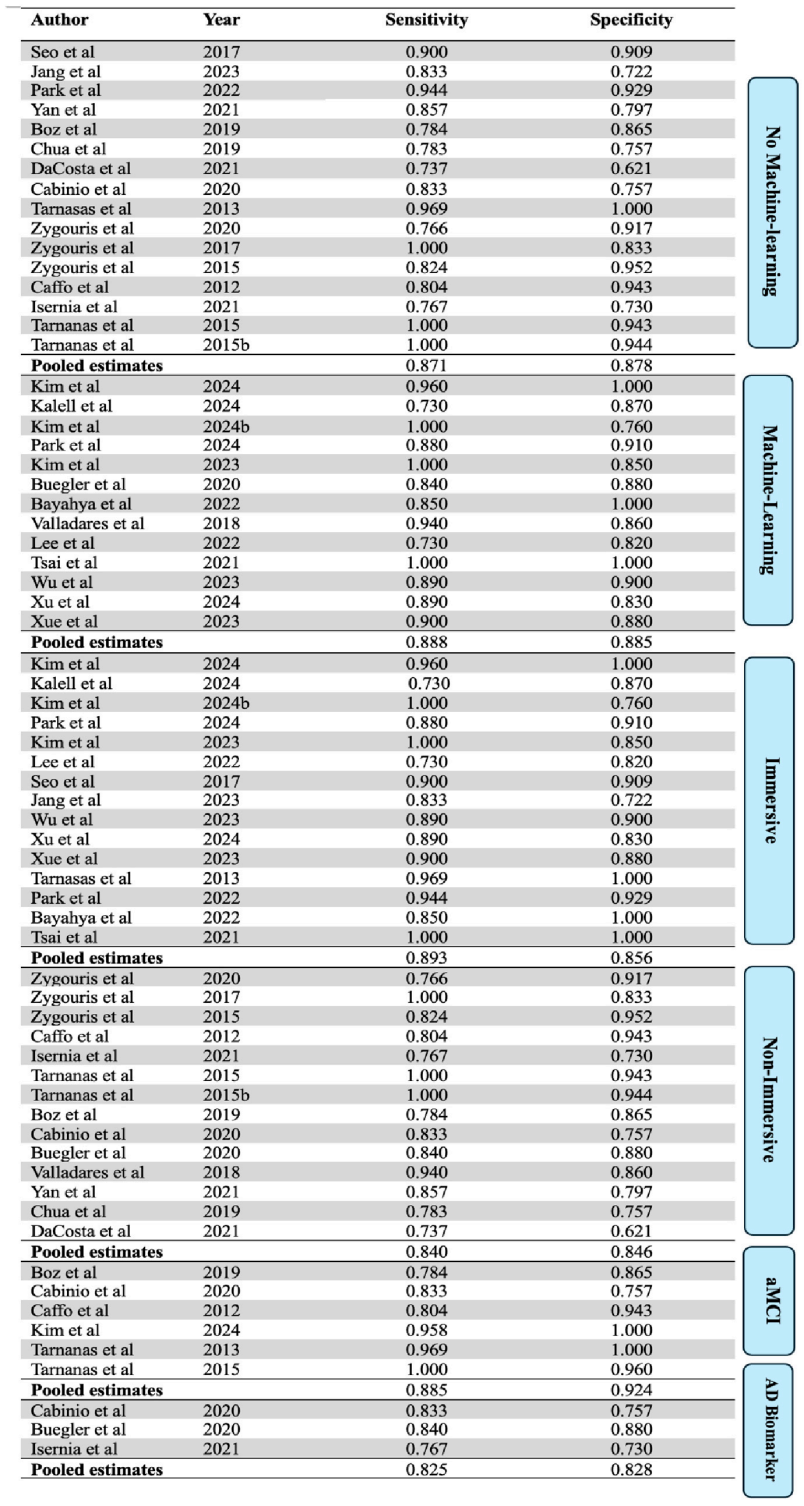
Sub group analysis. AD biomarker = studies that used AD biomarkers as a reference standard.

A subgroup analysis based on the immersion level of the assessment tools used in the included studies was also performed. Studies were classified as “immersive” if they used fully immersive technology, or “non-immersive” if they used semi- or non-immersive tools (see Figure 5). The 15 immersive studies showed a pooled sensitivity of 0.893 (95% CI: 0.855, 0.922) and a specificity of 0.856 (95% CI: 0.824, 0.884). The 14 non-immersive studies showed a pooled sensitivity of 0.840 (95% CI: 0.814, 0.867) and a specificity of 0.846 (95% CI: 0.820, 0.870).

Only three of the included studies used AD biomarkers (e.g., amyloid PET scans, CSF samples) as a reference standard. These studies showed a pooled sensitivity of 0.825 (95% CI: 0.779, 0.864) and a specificity of 0.828 (95% CI: 0.791, 0.859). When analyzing studies only including aMCI patients, the 6 aMCI studies yield a pooled sensitivity of 0.885 (95% CI: 0.753, 0.947) and specificity of 0.924 (95% CI: 0.776, 0.977).

### 3.5 Sensitivity analysis

For the sensitivity analysis, a one-by-one exclusion approach was applied, where each study was sequentially removed, and a meta-analysis was conducted on the remaining studies. The results indicated that excluding individual studies did not significantly alter the overall findings, suggesting that the meta-analysis results were relatively stable.

### 3.6 Analysis of publication bias

**Figure 6:**
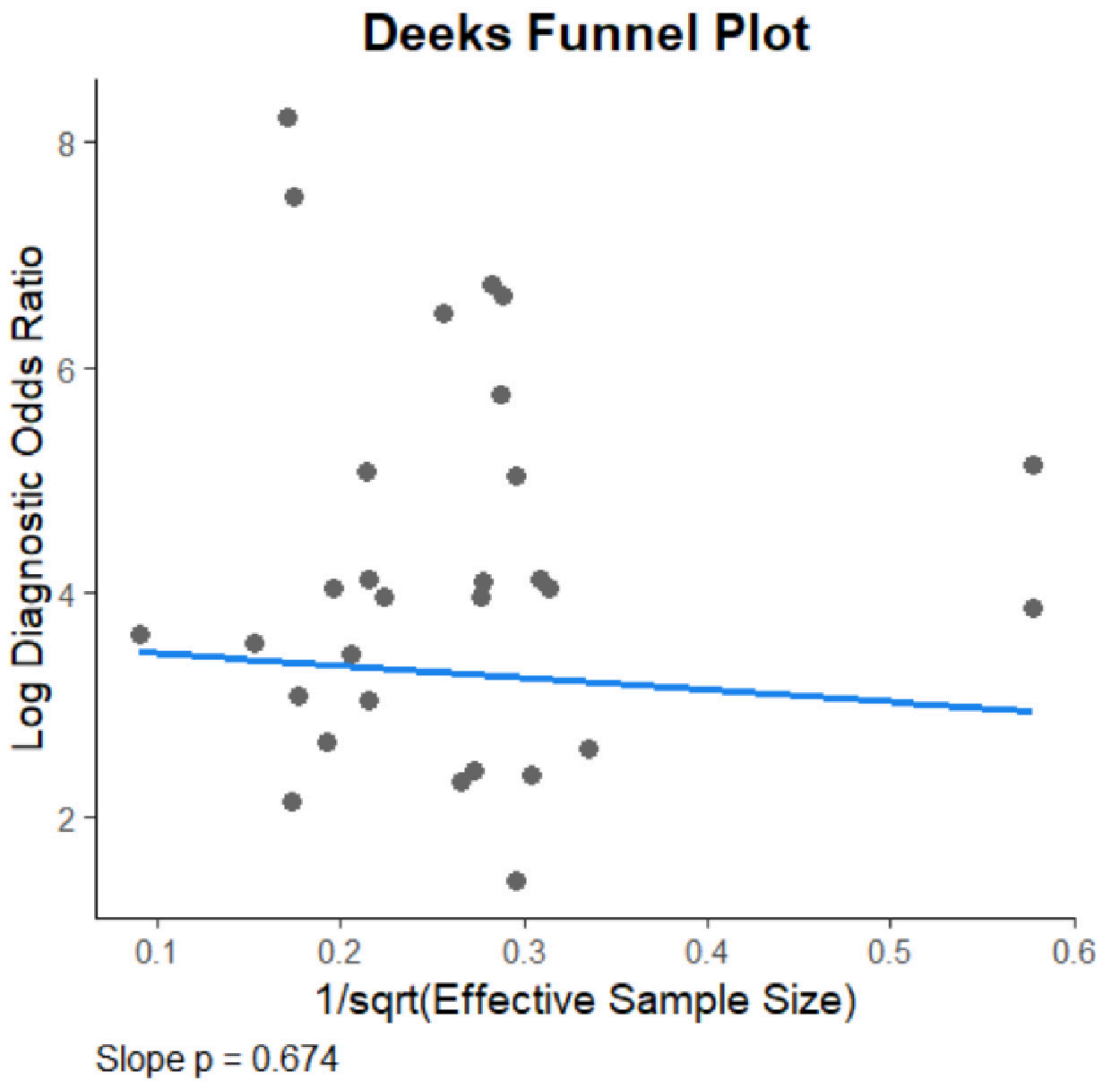
Deeks funnel plot.

The Deeks' funnel plot asymmetry test revealed no significant evidence of publication bias among the included studies (*P* = 0.674), indicating that the meta-analysis results are unlikely to be affected by selective publication (see Figure 6).

### 3.7 Risk of bias and quality assessment

The quality assessment was carried out following the methods described in the methods section. In the subsequent sections, the risk of bias for the included studies will be evaluated separately for those utilizing machine-learning and those that did not. This distinction is necessary because, as previously noted, the index test domain of the QUADAS-2 assessment tool is modified for machine-learning studies, which are subject to different types of biases compared to non-machine-learning studies.

#### 3.7.1 Machine-learning studies

**Figure 7:**
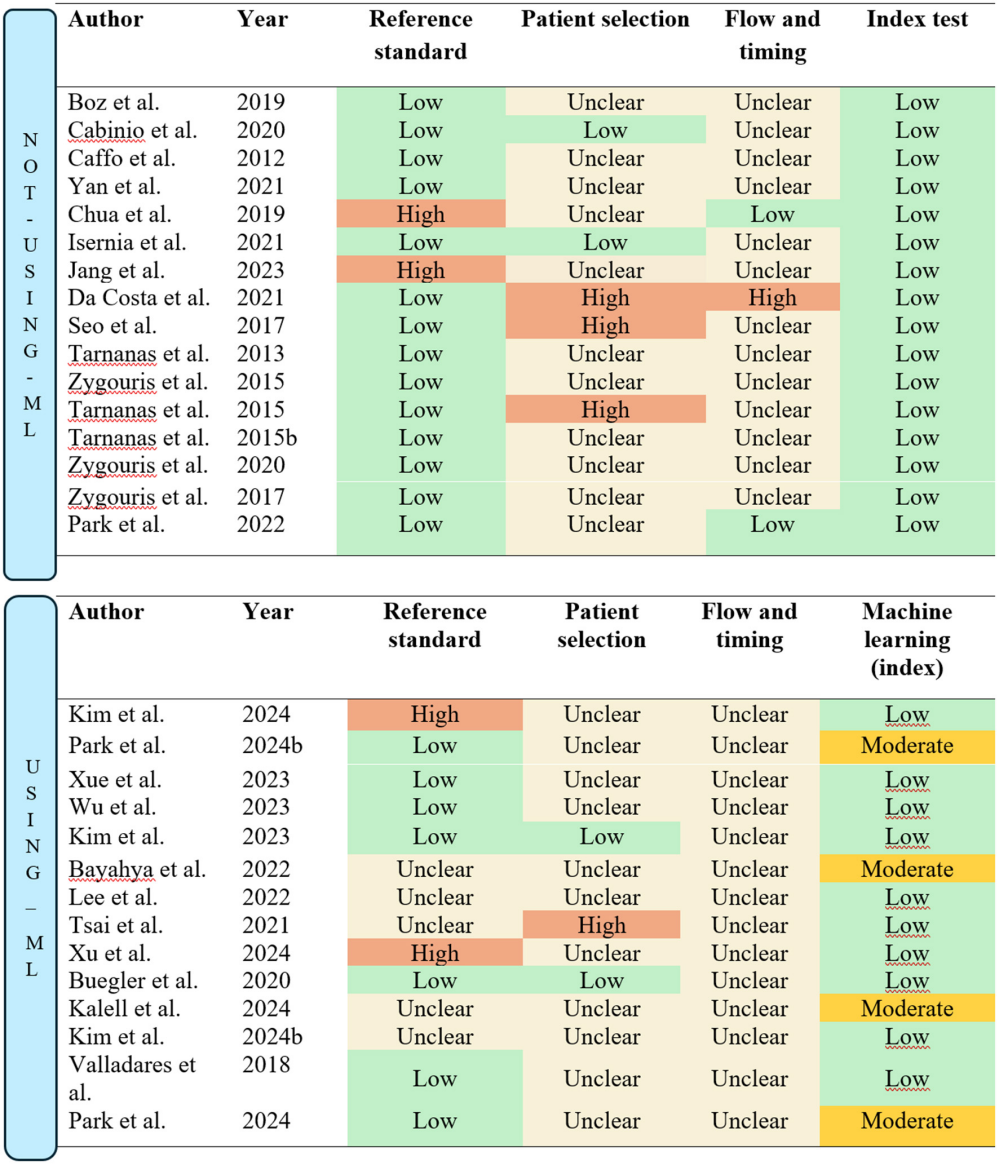
Quality assessment/risk of bias according to QUADS-2 guidelines. The figure is split into studies “Not using machine-learning (ML)” and “Using machine-learning (ML).”

The quality assessment, as summarized in Figure 7, was conducted using the QUADAS-2 domains. Most of the included studies utilized appropriate reference standards consistent with established guidelines for MCI diagnosis. However, five studies did not appropriately report specific criteria or tests used, resulting in an unclear classification for the reference standard domain (Bayahya et al., 2022; Kallel et al., 2024; Lee et al., 2022; Tsai et al., 2021; Kim S. Y. et al., 2024). Furthermore, Xu et al. (2024) was rated as high risk of bias due to only using the MoCA as the reference standard, while Kim S. Y. et al. (2024) was rated as high risk due to only using the Korean MMSE.

The patient selection domain was insufficiently described in most studies, particularly regarding the sampling methods. It was often unclear whether convenience sampling was avoided. Only two studies explicitly reported consecutive sampling and were thus classified as low risk in the patient selection domain (Buegler et al., 2020; Kim et al., 2023). Conversely, Tsai et al. (2021) was rated as high risk due to a lack of detail regarding the recruitment process.

In the flow and timing domain, none of the studies provided sufficient details on the time intervals between administering the reference standard and the index test. This lack of information resulted in all studies being classified as unclear for this domain.

For studies incorporating machine-learning methods, the validation process replaced the traditional index test domain in the QUADAS-2 assessment, as machine-learning-based tests require different considerations. Most studies employed cross-validation methods, which were rated as low risk. However, four studies used the holdout method, resulting in a moderate risk classification (Bayahya et al., 2022; Kallel et al., 2024; Kim S. Y. et al., 2024; Park et al., 2024).

#### 3.7.2 Studies not using machine-learning

For studies utilizing VR assessments without machine-learning, the conventional QUADAS-2 assessment criteria were applied. Most studies adhered to established guidelines and diagnostic standards for MCI, resulting in a low risk of bias in the reference standard domain (See Figure 7). Exceptions included Jang et al. (2023) and Chua et al. (2019), where the MoCA was the sole test used to classify participants. Additionally, all studies provided standardized and well-documented procedures for administering the index test, leading to a low risk of bias classification in the index test domain.

In the patient selection domain, only two studies (Cabinio et al., 2020; Isernia et al., 2021) were rated as low risk, as they explicitly described consecutive sampling methods. Conversely, three studies were assessed as high risk due to the use of a case-control design (Da Costa et al., 2021; Seo et al., 2017) or reliance on convenience sampling (Tarnanas et al., 2015a). The remaining studies were rated as having unclear risk due to insufficient detail regarding the recruitment process.

In the flow and timing domain, Da Costa et al. (2021) was rated high risk due to significant participant dropout, which may bias results, as those who drop out often differ significantly from those who remain. This loss of “real-world” variability compromises the representativeness of the sample. Furthermore, two studies were rated low risk, as they clearly outlined the timing between the reference standard and the index test (Chua et al., 2019; Park, 2022).

#### 3.7.3 Quality assessment summary

Overall, the quality assessment highlights poor reporting practices in many of the included studies, particularly in the flow and timing domain, where the majority were rated as unclear. This indicates that additional uncertainty should be considered, as the potential for bias in several studies remains indeterminate. Consequently, the results of the current meta-analysis should be interpreted with caution. While the statistical analysis suggests high certainty in the pooled estimates, the presence of unclear or unaddressed biases in the included studies introduces an additional layer of uncertainty to the findings.

## 4 Discussion

The findings of this study highlight the potential of VR-based assessments in detecting MCI and distinguishing it from healthy aging. By synthesizing data from multiple studies, this analysis provides insights into the diagnostic accuracy of VR tools and their integration with assistive technologies such as machine learning, EEG, and eye-tracking.

The current meta-analysis suggests that VR-assessments on average have a higher accuracy than the most common traditional pen-and-paper tests such as the Montreal Cognitive Assessment (MoCA) and the Mini Mental State Examination (MMSE). As an example, in a meta- analysis by Tsoi et al. (2015) it was found that the MMSE had a 62% sensitivity and 87% specificity in differentiating MCI from healthy controls. Furthermore, the MoCA was found to have an 89% sensitivity and 75% specificity. Slightly higher accuracy has been found for the MoCA in a meta-analysis by Carson et al. (2018), which found a sensitivity of 83% and specificity of 88%. When comparing the pooled sensitivity and specificity of VR assessments included in this meta-analysis, it appears that VR-assessments show great potential.

While the subgroup analysis indicated a slight accuracy advantage for machine-learning studies, it did not reveal a significant superiority over non-machine-learning methods. This is likely attributable to the exploratory and preliminary stage of current machine-learning research. Furthermore, most of the machine-learning studies were found to be more time-efficient, a topic that will be explored in greater detail. The subgroup analysis revealed a modest increase in accuracy for studies utilizing more immersive technology compared to non-immersive methods. Attributing this difference solely to the degree of immersion is challenging, as these studies also had more recent publication dates, incorporated more assistive technologies (e.g., EEG), and more frequently used machine learning. This suggests the observed increase in accuracy is likely multifactorial rather than a direct result of immersion alone.

In the 29 included studies there is great heterogeneity in the VR-tests used to distinguish MCI patients from healthy controls. The pooled accuracy estimates are therefore mostly informative on the level of assessing whether VR-assessments in general show potential to be accurate. However, it does not inform us directly which design-options for VR-assessments are the best. Moreover, the included studies were published between 2012 and 2024, meaning some may not fully reflect the current capabilities of VR-based assessments. One of VR's greatest strengths is its ability to integrate advanced technologies for large-scale, efficient data collection. The accessibility of these technologies has expanded only recently, driven by rapid advancements in AI and machine learning. The integration of machine learning into MCI diagnosis is a particularly recent development, as evidenced by the fact that all but one of the 13 machine learning studies included in this meta-analysis were published after 2020, with nine appearing since 2023. The findings of this meta-analysis should be interpreted within the context of VR-based assessments still being in their very early stages of development. Furthermore, time and cost-efficiency are also important factors to consider when comparing VR-assessments to traditional methods.

### 4.1 Time and cost-efficiency

Economically, dementia stands out as a primary reason for long-term institutional care in older adults, resulting in considerable healthcare costs (Wimo et al., 2011). Timely diagnosis of MCI is essential to provide appropriate drug and non-drug treatments, which can help slow disease progression and reduce rising healthcare expenses. An essential challenge lies in the methods used to diagnose MCI. Existing approaches are often either highly accurate but time-consuming, or relatively quick but less accurate, like the MMSE and MoCA. Virtual assessments, however, may have the potential to combine accuracy with time efficiency, making them a candidate for large-scale MCI screening.

#### 4.1.1 Time-efficiency

Time efficiency in testing is strongly linked to cost efficiency because shorter tests reduce the time required from healthcare professionals, leading to lower labor costs. Additionally, efficient tests enable higher patient throughput, maximizing the use of resources and minimizing delays. This is particularly important in large-scale screenings, where time savings probably translate directly into reduced operational expenses. Given that widely used screening tools like the MoCA and MMSE typically require 10–15 min to administer (Nasreddine et al., 2005; Tombaugh et al., 1996), virtual assessments should probably demonstrate comparable time requirements to be viable for widespread implementation. The studies included in this review highlight advancements in virtual assessments over the past decade, with feasibility primarily linked to more recently developed VR tools. These newer assessments incorporate the most advanced technologies and methodologies, making them better equipped to address the practical challenges of large-scale implementation, such as time efficiency and user accessibility. Among the included studies, ten report time to completion, all of which fall under 30 min (see Table 6). Notably, assessments such as the Virtual Kiosk Test, VECA, Altoida DNS, and VRNPT demonstrate time-efficiency comparable to or exceeding that of traditional tools like the MoCA and MMSE (all of which are assisted by machine-learning).

**Table 6:** Included studies that reported time to completion.

**Author**	**Assessment tool**	**Time to completion**	**Self-administered**
Xue et al. (2023)	VRNPT	17 min	No
Kim et al. (2023) Kim D. et al. (2024) Kim S. Y. et al. (2024) Park et al. (2024) Kallel et al. (2024)	Virtual kiosk test	5–15 min	No
Buegler et al. (2020)	Altoida DNS	10 min	Yes
Xu et al. (2024)	VECA	5 min	Yes
Lee et al. (2022)	N/A	Less than 30 min	No
Jang et al. (2023)	VARABOM	19 min	No
Eraslan Boz et al. (2019)	Virtual supermarket program	25 min	No
Chua et al. (2019)	REACH	19–20 min	No
Zygouris et al. (2020)	Virtual supermarket program	30 min	Yes
Zygouris et al. (2015)	VAP-M	30 min	No

Another strategy for enhancing the feasibility of MCI screening is to enable self-administration. If assessments can be conducted remotely or independently by users, time efficiency becomes less critical, as this approach minimizes the need for direct clinician involvement. Three of the included studies (Buegler et al., 2020; Xu et al., 2024; Zygouris et al., 2020) specifically designed their VR assessments for self-administration. These tools have the potential to significantly reduce the time clinicians spend on screening and lower the reliance on specialized personnel for MCI diagnosis. The Altoida DNS, in Buegler et al. (2020), is notable for its focus on longitudinal screening of MCI and Alzheimer's disease. It has been specifically tested for repeated self-administrations in a home setting, making it an effective tool for ongoing monitoring and early detection.

#### 4.1.2 Cost efficiency

Self-administration and time efficiency are key factors in reducing MCI screening costs, as much of the expense arises from the time required by trained professionals (Michalowsky et al., 2017; Wimo et al., 2024). Several VR assessments, as noted previously, are seemingly more time-efficient than traditional tools like the MoCA and MMSE and support self-administration. However, concerns remain about the expenses associated with VR technology. Interviews with health practitioners reveal that implementation costs and associated technologies are seen as significant barriers (Yondjo and Siette, 2024). Uncertain and potentially high costs for software, machine-learning capabilities, and assistive devices such as EEG systems, eye-tracking tools, and VR headsets add to these challenges.

However, these technologies are becoming increasingly affordable. For instance, the Muse-2 EEG headband, utilized in VR assessments by Xu et al. (2024) and Wu et al. (2023), offers a low-cost solution priced between 187 and 232 USD (InteraXon Inc., n.d.) and integrates seamlessly with VR platforms. Similarly, many commercially available VR headsets now include built-in eye-tracking and motion sensors. Devices like the SAMSUNG HMD Odyssey+ are becoming more accessible, with a consumer price of approximately 299 USD (Amazon, n.d.). Accurate and time-efficient virtual assessments could provide significant value by reducing costs in the diagnostic process. As disease-modifying therapies will likely target patients with molecular evidence of dementia (e.g., Alzheimer's), virtual tools can streamline referrals to expensive methods like MRI or CSF sampling. Current diagnostic techniques, such as MRI, CSF analysis, and PET, incur substantial costs per session. Avoiding a single false-positive during screening could save 500–1,000 euros or more per patient (Wimo et al., 2024). Thus, investing in VR-based assessments may offer an economically viable solution to minimize the financial burden of misdiagnoses. Cost-efficiency analyses indicate that even modestly effective disease-modifying therapies for dementia could be a financially sound investment, helping to alleviate the long-term economic burden of dementia (Green et al., 2019). Wittenberg et al. (2019) estimate that 100,000 additional amyloid PET scans in the UK would cost £113 million, while CSF testing would add £48 million. Despite these costs, they argue that molecular testing expenses are outweighed by the potential benefits of effective Alzheimer's treatments.

Accurate MCI-screening can therefore optimize molecular testing by minimizing incorrect referrals and ensuring appropriate use, reducing diagnostic costs. Nevertheless, concerns remain regarding the technical expertise required to operate and maintain systems combining machine learning and assessment software. Virtual assessments, while promising, present a complex and potentially costly alternative to traditional methods, especially when they incorporate advanced technologies. For instance, VR-based assessments that utilize machine learning and multiple data types (such as EEG or speech metrics) will likely require a high level of specialized knowledge. In their current state, these systems demand that clinicians be adept at handling the collection, preprocessing, synchronization, and interpretation of diverse data from machine learning models. Although some software, like the Altoida DNS, streamlines this process with user-friendly interfaces, more complex systems that integrate multiple data modalities like EEG, motor kinematics, and speech metrics will likely require extensive personnel training. Furthermore, an increase in data modalities also increases the likelihood of technical malfunctions, necessitating specialized staff to manage these issues. The total costs are therefore uncertain and potentially high, stemming not only from the need for specialized personnel but also from expenses related to data security, software licensing, and technical support.

### 4.2 Acceptance among older populations

While VR-based assessments offer several potential advantages, their implementation also presents certain challenges. In particular, the adoption of VR assessments among older populations raises several concerns. One is that of cybersickness (motion sickness) often experienced especially by older populations when using certain immersive types of VR (Margrett et al., 2022), another is familiarity and attitudes toward interacting with new technologies and whether this form of assessment is less/more enjoyable than conventional pen and paper tests.

Seven studies in this review also examined participants' experiences, attitudes, and acceptance of VR technology, though their primary focus was on accuracy. In this case, Xue et al. (2023) reported a 94.7% satisfaction rate for the VRNPT, citing ease of use and intuitive design. Similarly, Zygouris et al. (2015, 2017) demonstrated that participants, including those with limited education, could independently complete the Virtual Supermarket (VSM) test without major technical issues. Jang et al. (2023) found no dropouts due to cybersickness or usability challenges, with intuitive interfaces improving user comfort. Cabinio et al. (2020) reported successful completion across varying levels of prior computer experience, while Chua et al. (2019) observed a 100% completion rate and high user satisfaction. Eraslan Boz et al. (2019) noted greater engagement with VR tasks compared to traditional tests. Overall, these findings suggest that older adults generally find VR assessments engaging, feasible, and user-friendly. While some participants reported minor usability issues, the studies highlight VR's potential as an accessible cognitive assessment tool. However, as these studies primarily assessed accuracy, more rigorous research on acceptance in older populations is needed.

When looking at relevant studies that only look at acceptance among older populations, these results seem to be corroborated, however; acceptance is found to differ with education and culture. For instance, A study by Siette et al. (2024) reported that 78% of participants were willing to use VR applications for cognitive screening, though only 24.7% expressed willingness to engage with them weekly. Higher acceptance rates were observed among participants with greater educational attainment, suggesting that familiarity with technology and education significantly influence acceptance. Cultural factors also play a role; Mondellini et al. (2022) compared Italian and Estonian older adults with MCI and found higher acceptance and a stronger sense of presence among Italians. Differences in attitudes toward technology, rather than physical reactions like cybersickness, were identified as the primary reason for these discrepancies. In summary, while older adults generally seem to have a positive perception of VR screening tools, personal, cultural, and educational factors can hinder widespread adoption and frequent use. Encouragingly, users report few side effects, such as cybersickness, especially when physical movement within VR environments is restricted. Moreover, future generations of older adults are likely to exhibit greater familiarity with and acceptance of VR technology.

### 4.3 Future directions for the application of technologies in VR assessment

Many of the included studies in the current study, employ assistive technologies, that are integrated with the different VR-assessment routines. Each technology may offer unique insights into cognitive function, yet their combined application within VR may yield a more comprehensive and accurate assessment. This section explores the synergistic potential of these assistive tools within a unified VR framework, while identifying the most promising avenues for future development. By understanding the strengths of each technology, we can better inform the design of next-generation VR tests for effective MCI detection.

A minority of the included studies assess the individual contributions of these assistive technologies. Among the included studies, the use of EEG has the most data on its standalone effects, while the independent impact of eye-movement data was examined in only one study. Of all assistive technologies, motion tracking was the most frequently used assistive technology and showed promising results, though only one study (Kim et al., 2023) reported the impact of movement data as an independent variable. Nevertheless, some of the studies rely heavily on movement data and report high accuracies. For instance, the DNS in Buegler et al. (2020) employs 109 motor behaviors for assessment, with only four variables (time to hide object, time to find object, location errors, and order errors) unrelated to movement, suggesting that movement data significantly contributes to accuracy.

Additionally, the Virtual Kiosk Test, used in five of the included studies (see Table 7), integrates movement as a core assessment component alongside eye-movement, time to completion, and number of errors. Eye-tracking appears to contribute modestly to the Virtual Kiosk Test's accuracy (60% accuracy, 100% sensitivity, 0% specificity), while movement data as an independent variable achieved 88.9% sensitivity and 66.7% specificity (80% accuracy). It is likely that movement data similarly contributes to accuracy across the other five studies using the Virtual Kiosk Test, as high accuracies were consistently observed.

**Table 7:** Combinations of assistive technologies used in the virtual kiosk test and accuracy.

**Virtual kiosk test**							
Assistive technology		Kim et al., 2023	Kim D. et al., 2024	Kim D. et al., 2024	Park et al., 2024	Kallel et al., 2024	
VR		93,3%	N/A	88,24%	88,9%	82,53%	
EEG		N/A	93,33%	88,24%	N/A	74%	
MRI		N/A	N/A	64,71%	83,3%	82,35%	
VR+MRI		N/A	N/A	N/A	94,4%	N/A	
VR+EEG		N/A	98,38%	N/A	N/A	N/A	
VR+EEG+MRI		N/A	N/A	94,12%	N/A	86,66%	
**Other studies using assistive technologies**							
Assistive technology	Tarnanas et al., 2013	Seo et al., 2017	Buegler et al., 2020	Lee et al., 2022	Wu et al., 2023	Xue et al., 2023	Xu et al., 2024
VR	N/A	N/A	N/A	75,8%	79%	N/A	N/A
Speech	N/A	N/A	N/A	N/A	81%	N/A	N/A
EEG	N/A	N/A	N/A	65,6%	83%	N/A	N/A
VR+EEG	N/A	N/A	N/A	80%	83%	88,7%	N/A
VR+EYE	N/A	N/A	N/A	N/A	N/A	N/A	85,75%
VR+MVMT	98,5%	92,9%	86%	N/A	N/A	N/A	N/A
VR+SPEECH+EEG	N/A	N/A	N/A	N/A	89,8%	N/A	N/A

MVMT = movement data and EYE = eye-movement data.

Three of the included studies also used MRI as an assistive technology, however MRI is not feasible to measure concurrently with VR-assessment. A justification for using 60–90 min on MRI acquisition (Kallel et al., 2024; Park et al., 2024) must be a large increase in accuracy and near perfect detection capabilities, however this was not illustrated in the included studies (see Table 7). A separate table was created specifically for the Virtual Kiosk Test, as numerous studies focus exclusively on how assistive technologies enhance and complement this assessment method. Combinations of assistive technologies in tests other than the virtual kiosk test are illustrated in the bottom part of Table 7, under “other studies using assisitive technologies”.

EEG and movement-data emerge as the assistive technologies with the thus far, strongest results. While EEG may have the highest individual contribution to test accuracy, it seems harder to implement with VR-testing than movement data, as interference could be an issue. Moreover, only three of the six EEG-studies used EEG simultaneously with VR assessment, while all (9) movement-data studies recorded movement data simultaneously with VR-assessment. This might suggest movement data is easier to implement in VR-assessment compared to EEG. The collection of speech data showed strong results in Wu et al. (2023); however, there is limited data, as Wu et al. (2023) was the only study incorporating this approach. Additionally, the study lacked information on the time efficiency of collecting speech data. Nonetheless, given that speech data collection is automated through wearable devices, this approach holds promise for integration into future VR assessments.

Given the accuracy and time-efficiency considerations of these technologies, eye-movement data collection could plausibly be excluded from the virtual kiosk test, replaced by lightweight, wearable EEG technologies like the Muse 2 used in Wu et al. (2023) and Xue et al. (2023). Kim et al. (2023) reported that the virtual kiosk test takes approximately 5 min, while practice and eye calibration sessions extend the time by about 10 min, with calibration alone taking around 7 min. Since eye-tracking contributes a specificity of 0%, omitting it could increase time-efficiency without major impacts on accuracy. Incorporating a brief scene description after the test could add valuable speech data. Although no studies to date have combined EEG with movement data in VR, a study by Chai et al. (2023) achieved 96.3% accuracy in detecting MCI by analyzing handwriting dynamics alongside EEG in a machine-learning study. Future research could explore a multi-modal approach integrating motor, EEG, and speech data in a virtual assessment, ideally within a 10–20-min timeframe.

When tests are self-administered, time-efficiency might be less of a concern as the cost of trained professionals seemingly creates the largest barriers for large-scale screening. Therefore, beyond accuracy and time efficiency, there is an increasing focus on enabling self-administered screenings that do not require a visit to a hospital or clinic. The future of virtual assessments may therefore be the use of assessments that can be self-administered from home, while overseen by professionals. As an example, the DNS by Buegler et al. (2020) illustrated that motor data can be collected in self-administered tests with high accuracy in differentiating healthy controls, MCI and AD. The DNS's time efficiency, self-administration, and strong predictive capabilities have led to its FDA “Breakthrough Device” status (Park, 2021). This designation accelerates development and regulatory review processes for technologies with the potential to significantly impact patient outcomes, underlining the potential of implementing self-administered tests. Furthermore, although not meeting the inclusion criteria of this review, Yamada et al. (2023) has illustrated the feasibility of a mobile app with automatic speech analysis for self-administered early AD and MCI screening, achieving 87.6% accuracy in differentiating healthy controls from MCI. Analyzing both motor and speech data could increase the accuracy of future self-administered tests.

The diagnostic value of VR assessments is likely due to the individual data modalities, such as EEG and motor kinematics, rather than the level of immersion. This is supported by the similar diagnostic accuracies found in both fully immersive and non-immersive VR assessments. It is likely that fully immersive VR headsets may not be essential for effective screening. Instead, digital or non-immersive VR technology could still be beneficial for the efficient and simultaneous collection of multiple data modalities. Moreover, the future of dementia screening may not need to rely on VR technology at all. Data such as speech metrics, movement patterns, EEG, and eye-tracking could be collected independently or in combination without a VR interface. For example, Yamada et al. (2023) demonstrated the successful use of speech metrics without VR to differentiate healthy individuals from those with MCI. Similarly, analyses of handwriting kinematics using digital pens have shown comparable accuracy to some VR-based assessments (Garre-Olmo et al., 2017; Nardone et al., 2025). However, it is likely that some sort of non-immersive VR-technology will ease the simultaneous collection of multiple modalities. This highlights that while VR-based assessments are a key focus, other promising, technologically enhanced methods for dementia screening exist. A common thread among these innovative approaches is their reliance on machine learning to analyze the collected data. Future research should investigate how the simultaneous collection of EEG, speech, and motor data could improve diagnostic accuracy.

### 4.4 Limitations

#### 4.4.1 Inaccurate reference standards

An important limitation in evaluating the accuracy of diagnostic tools in this meta-analysis is the reliance on reference standards that may themselves be flawed or less accurate than the index tests being assessed. The performance of an index test is typically evaluated by comparing its results to a reference standard, often considered the gold standard for diagnosis. However, in the context of MCI, many of these reference standards are not without their limitations, raising critical concerns about the validity of comparing innovative diagnostic tools to benchmarks that may not be sufficiently reliable. Current reference standards, such as neuropsychological test batteries and AD biomarkers may be prone to several shortcomings. When an index test is evaluated against a flawed reference standard, its true accuracy can be misrepresented, particularly when the test is more sensitive or specific than the reference standard. For example, an index test may correctly detect early-stage MCI cases that the reference standard misses, leading to true positives being mislabeled as false positives. This challenge is particularly relevant for advanced diagnostic tools, such as machine learning-assisted assessments, which can identify subtle cognitive changes—like variations in EEG signals, eye movements, motor functions, or speech—that may go undetected by current gold-standard testing procedures. This dynamic creates a broader challenge in the development and evaluation of highly accurate diagnostic tests. As these tools advance in their ability to detect subtle cognitive changes, they may surpass the diagnostic capabilities of the current gold standard, making direct comparisons problematic. The paradox lies in the fact that the potential of these innovative tools may be constrained by the limitations of the very benchmarks used to validate them. The use of imperfect reference standards in some of the included studies may have introduced bias into the meta-analysis, potentially affecting its ability to accurately reflect the true diagnostic performance of VR-based assessments. Validation studies should therefore move beyond current gold standards and assess diagnostic tools against long-term clinical outcomes, which can offer a more reliable measure of their accuracy and predictive value.

This relates to the lack of longitudinal studies among the included research. Cross-sectional studies are typically favored in the initial phases of test development because they are faster and less resource-intensive than longitudinal studies. These designs focus on evaluating the agreement between the index test and an accepted reference standard rather than its ability to predict long-term outcomes. The predominance of cross-sectional studies underscores how preliminary this area of research still is, particularly when examining the integration of VR technologies with advanced methods such as machine learning. When validating assessment tools that have the potential to surpass the accuracy of current gold standards, independent validation studies focusing on long-term clinical outcomes are particularly crucial. Such studies can mitigate the biases introduced by flawed reference standards by directly linking the test's predictions to meaningful, real-world outcomes.

Only three studies employed a longitudinal design to evaluate the accuracy of VR-assessments in predicting dementia progression over time (Buegler et al., 2020; Tarnanas et al., 2013; Zygouris et al., 2017). Among these, Zygouris et al. (2017) included only 12 participants, which significantly limits the reliability and generalizability of its findings. Tarnanas et al. (2013) conducted a moderately sized study with 205 participants, while Buegler et al. (2020) undertook a large international, multi-center study involving 496 participants across multiple countries. The most compelling evidence to date regarding the longitudinal validity of VR-based assessments comes from the study by Buegler et al. (2020). This study demonstrated that a VR-based tool could predict conversion to Alzheimer's disease (AD) with 94% accuracy, achieving 88% specificity and 84% sensitivity for identifying MCI over a 60-month period. These results are promising and suggest that VR assessments have significant potential for predicting long-term cognitive outcomes. However, conclusions about the overall accuracy and longitudinal validity of VR-based assessments remain constrained by the predominance of cross-sectional studies included in this meta-analysis. To advance the field, future research must move beyond the limitations of cross-sectional designs and prioritize longitudinal studies that evaluate the predictive validity of these tools over time.

#### 4.4.2 Heterogeneity and limitations to generalizability

Another limitation is the heterogeneity in the included studies. These differences can impact the comparability of the studies and influence the overall conclusions that can be drawn from the meta-analysis. The current meta-analysis examines VR-based assessments as a broad, umbrella term. However, these assessments differ significantly in their specific characteristics, as well as in how they are designed and conducted. Each VR assessment varies in critical aspects, such as the cognitive domains they target, the tasks participants perform within the virtual environment, and the technologies they incorporate. The included studies also varied in their focus, with some targeting amnestic MCI and others examining just MCI. Since aMCI primarily involves memory deficits, it may be easier to design accurate tests for this subtype compared to general MCI, which spans multiple cognitive domains. This variation likely contributes to heterogeneity in the meta-analysis. Additional heterogeneity arises from differences in study design, including varying levels of VR immersion, from highly immersive setups to simpler ones like touch screens. Many of the included studies, particularly those involving machine learning, originated from Asian countries, with South Korea contributing the largest number. While a considerable number of studies were conducted in European countries, the findings of this meta-analysis are likely more applicable to Asian populations than to those in Europe or North America. Finally, the studies included in this meta-analysis used different reference standards to validate the VR assessments. While some relied on comprehensive neuropsychological evaluations or biomarkers, others used less rigorous approaches such as using the MoCA as the only reference standard. When generalizing these results to Alzheimer's disease (AD), caution is necessary. Only three of the included studies (Buegler et al., 2020; Cabinio et al., 2020; Isernia et al., 2021) used reference standards that directly targeted AD biomarkers, which are considered the current gold standard for diagnosing MCI due to AD (Jack et al., 2024). This inconsistency in reference standards introduces potential bias, complicating comparisons of diagnostic accuracy across studies. As a result, the pooled accuracy metrics should be interpreted with caution, as they represent an exploratory overview rather than a true average of the diagnostic accuracy of VR-based assessments for MCI.

## 5 Conclusion

The pooled accuracy estimates from this meta-analysis indicate that VR-based assessments collectively demonstrate high diagnostic accuracy. When compared to widely used screening tools like the MMSE and the MoCA, the findings suggest that VR-based assessments exhibit higher accuracy than the MMSE and comparable or slightly higher accuracy than the MoCA on average. However, the conclusions are limited by methodological issues in the included studies, suggesting a high potential for bias in the analysis. Furthermore, some VR assessments were found to be more time-efficient than traditional methods. In terms of integration with other technologies, EEG and movement analysis stand out as key contributors to diagnostic accuracy and may be well-suited for integration with VR environments. With machine-learning algorithms, VR assessments can efficiently process large datasets. As the field evolves, integrating these technologies holds promise for improving the accuracy and efficiency of VR-based assessments. Beyond accuracy, VR assessments also show promise in terms of feasibility. However, clinical implementation may face notable barriers, including the requirement for specialized personnel and the absence of clear data regarding software and support costs.

## Data Availability

The original contributions presented in the study are included in the article/supplementary material, further inquiries can be directed to the corresponding author.
